# The effect on income of providing near vision correction to workers in Bangladesh: The THRIVE (Tradespeople and Hand-workers Rural Initiative for a Vision-enhanced Economy) randomized controlled trial

**DOI:** 10.1371/journal.pone.0296115

**Published:** 2024-04-03

**Authors:** Farzana Sehrin, Ling Jin, Kamrun Naher, Narayan Chandra Das, Ving Fan Chan, Dong Feng Li, Susan Bergson, Ella Gudwin, Mike Clarke, Tai Stephan, Nathan Congdon

**Affiliations:** 1 BRAC (Formerly: Bangladesh Rural Advancement Committee), Dhaka, Bangladesh; 2 State Key Laboratory of Ophthalmology, Zhongshan Ophthalmic Center, Sun Yat-sen University, Guangzhou, China; 3 University of KwaZulu Natal, Durban, South Africa; 4 Centre for Public Health, Queen’s University Belfast, Belfast, Northern Ireland, United Kingdom; 5 VisionSpring, New York, New York, United States of America; 6 Orbis International, New York, New York, United States of America; Democritus University of Thrace, GREECE

## Abstract

**Introduction:**

Presbyopia, the leading cause of vision impairment globally, is common during working years. However, no trials have assessed presbyopia’s impact on income.

**Methods:**

In April 2017, we conducted a census among 59 Bangladesh villages to identify persons aged 35 to 65 years with presbyopia (presenting distance vision > = 6/12 bilaterally and correctable inability to see 6/13 at 40 cm with both eyes), who never had owned glasses. Participants were randomized (1:1) to receive immediate free reading glasses (intervention) or glasses delivered 8 months later (control). Visual demand of different jobs was stratified into three levels. Outcomes were between-group differences in the 8 month change in: self-reported monthly income (primary) and Near Vision Related Quality of Life (NVRQOL, secondary).

**Results:**

Among 10,884 census participants, 3,655 (33.6%) met vision criteria and 863 (23.6%) comprised a sample enriched for near vision-intensive jobs, but 39 (4.52%) could not be reached. All participants allocated to intervention (n = 423, 51.3%) and control (n = 401, 48.7%) received the appropriate intervention, and follow-up was available for 93.4% and 96.8% respectively. Groups were similar at baseline in all characteristics: mean age was 47 years, 50% were male, 35% literate, and about half engaged in "most near vision-intensive" occupations. Glasses wear at 8-month follow-up was 88.3% and 7.81% in intervention and control respectively. At baseline, both the intervention and control groups had a self-reported median monthly income of US$35.3. At endline, the median income for the intervention group was US$47.1 compared with US$35.3 for control, a difference of 33.4%. Predictors of greater income increase in multivariate models included intervention group allocation (OR 1.45, 95% CI 1.12, 1.88, P = 0.005), male sex (OR 2.41, 95% CI 1.84, 3.16, P <0.001), and not engaging in income-producing work at baseline (OR 2.35, 95% CI 1.69, 3.26, P<0.001).

**Conclusion:**

Provision of reading glasses increases income in near vision-intensive occupations, and may facilitate return to work for those currently unemployed.

## Introduction

Presbyopia, an age-related progressive decline in the unaided ability to see near objects [1], commonly manifests around age 40, and is essentially complete by age 55 [2]. Presbyopia is the most common cause of visual impairment, affecting 1.8 billion people globally [3]. Though presbyopia is safely, effectively, and inexpensively corrected with eyeglasses, uncorrected presbyopia impairs an estimated 826 million people’s ability to perform routine near tasks [3], among whom more than 90% live in low and middle-income countries (LMICs) [4]. The most recent data from Bangladesh found only 3.2% of persons with presbyopia had correction [5] a figure typical of LMICs.

Cross-sectional and prospective studies from LMICs suggest uncorrected presbyopia affects various economic activities, including reading, writing, cooking, use of mobile phones and other tools, sewing, weeding, and recognising money [6, 7]. PROSPER (PROductivity Study of Presbyopia Elimination in Rural-dwellers), the first randomised trial on the effect of presbyopia correction on workplace productivity, found that providing inexpensive reading glasses improved the productivity of tea pickers in Assam, India, by 21.7% [8]. This effect size is larger than reported for trials of other health-related interventions on work output [8]. The potential to improve productivity and income of older workers is of interest due to the global population’s rapid aging and declining rates of workforce participation in LMICs among persons over age 45 [9].

While evidence suggests providing reading glasses to presbyopic workers may be an attractive poverty alleviation strategy, advancing the first Sustainable Development Goal (SDG), evidence gaps remain. The PROSPER trial tested the impact of presbyopia correction in only a single work setting; did not directly assess effects on income; and used experienced optometrists to determine the power of the reading glasses provided [8]. Such highly-trained personnel are unavailable in many low-resource settings. The current trial fills evidence gaps by testing the impact of presbyopia correction in multiple near vision-intensive occupations, reporting a primary outcome of change in self-reported income, and by using non-medical personnel, who first underwent a brief training, to determine the prescription power of reading glasses.

We hypothesized that providing reading glasses would result in increased income and near-vision-related quality of life (NVRQOL) [10] among presbyopic participants ages 35 to 65 in this setting. Our primary objective is investigating whether the provision of free reading glasses for presbyopia correction can increase the income of persons pursuing visually-intensive occupations in Bangladesh, and the secondary objective is to determine whether the provision of free reading glasses can improve participants’ quality of life.

## Materials and methods

The THRIVE (Tradespeople and Hand-workers Rural Initiative for a Vision-enhanced Economy) investigator-masked randomised controlled trial was carried out in 59 villages across 15 districts in Bangladesh. These villages were purposively selected for having not been served previously by the Reading Glasses for Improved Livelihoods Program. Through this program, implemented by VisionSpring and BRAC (formerly: Bangladesh Rural Advancement Committee) since 2006, 25,000 community health workers in 61 of 64 districts in Bangladesh conduct vision screening, dispense reading glasses and refer for other ocular conditions. The protocol was approved in advance by the ethics committee at BRAC, Dhaka, Bangladesh. Oral informed consent was obtained from all participants before enrolment, and the tenets of the Declaration of Helsinki were followed throughout. The trial was registered at ClinicalTrial.gov (NCT03719196). Although ethics approval was received prior to enrolment of the first participant, due to an administrative error, responses to requested changes were not made and registration was not completed on the ClinicalTrials.gov website until after patient enrolment was begun. This study and all related trials are fully registered on ClinicalTrials.gov. The study protocol is available online at: https://www.qub.ac.uk/research-centres/CentreforPublicHealth/Research/HealthServicesGlobalHealth/EyeandVision/THRIVE/.

### Participants

**Fig 1:**
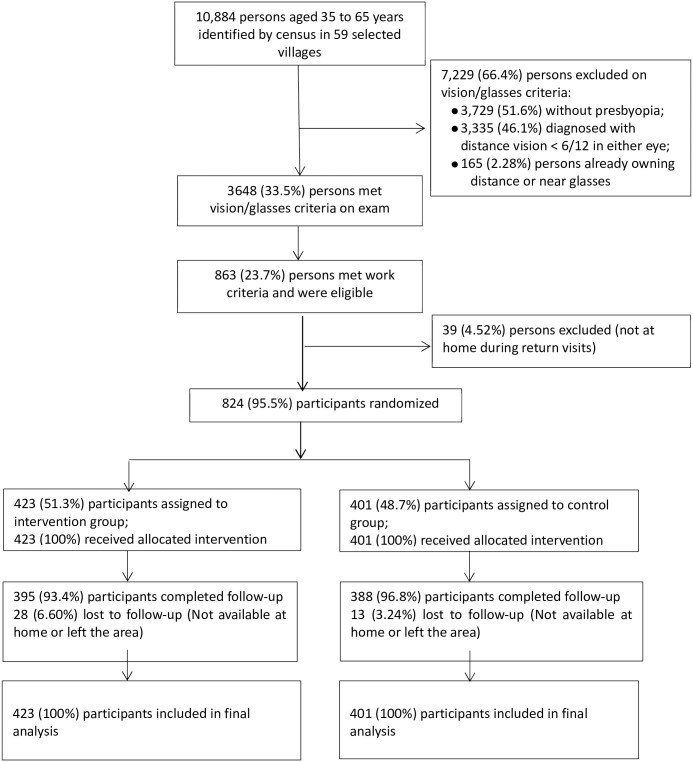
Flowchart depicting enrolment and progress of participants through the trial.

In April 2017, a census of the selected 59 villages was carried out to identify all persons aged between 35 and 65 years, who were invited to give consent and undergo screening of vision, eye health and other eligibility criteria by teams of non-medical data collectors. Inclusion criteria were: lack of apparent ocular conditions under gross, external examination under natural light; usual participation in paid occupations requiring intensive close work (such as tailors, artisans, mechanics, carpenters, farmers and shopkeepers); uncorrected distance visual acuity 6/12 or better in each eye; presence of presbyopia (defined as inability to see the 6/13 optotype at 40 cm with both eyes together, correctable with near vision glasses), and never having used near or distance glasses. Participants could be temporarily unemployed at the time of enrolment. Persons aged under 35 or over 65 years or having a self-reported history of serious medical, mental and/or ocular disease were excluded. To avoid contamination between the study groups, we randomly selected one person per household (Fig 1). In order to complete the target sample size, it was also necessary to include a moderate number (432, 52.4% of the total sample) of persons with less near vision-intensive occupations, resulting in a study cohort enriched for visually-demanding work.

### Randomisation and intervention

Eligible persons were randomly assigned 1:1 on a household basis (with only one participant per household) to immediately receive free near vision glasses providing best-corrected near vision with the two eyes together at a distance of 40cm (intervention group) or delivery of identical glasses after an 8-month observation period (control). Based on the census, door-to-door visits was conducted to get the data of persons over and above of 40 years of age. We received a pool of eligible participants for the study. There was random selection for the treatment and control arm using random numbers generated by Stata software and kept in sequentially-numbered opaque envelopes. So randomization was done for all participants based on sequentially-ordered envelopes in order to maintain concealment, and this was done in large batches to avoid the field workers having to carry large numbers of envelopes.

## Procedures

### Ocular and vision assessment and selection of glasses power

In August 2017, after three days of training by a community health specialist and an ophthalmologist, non-medical data collectors measured distance visual acuity separately in each eye for all persons identified in the census aged 35–65 years, and without obvious ocular abnormality as defined above. A modified Snellen Tumbling ‘E’ chart, marked with only a single optotype on each of the 6/6 and 6/12 lines, was used at a distance of 3 metres in a well-lit indoor or outdoor area of the participant’s home. Participants able to correctly read the 6/12 optotype with both eyes separately at 3 m were deemed to have satisfied the distance vision eligibility criterion and next underwent assessment of unaided near vision with both eyes simultaneously at 40 cm.

Near vision was assessed using a tumbling ‘E’ chart designed for use at 40 cm. Unaided near vision was determined by having the subject read the optotypes on the chart at 40cm without glasses and with both eyes open. Unaided near vision was recorded as the smallest line in which all optotypes were correctly identified. Those who were unable to correctly identify the 0.8M optotypes (the 40cm equivalent of 6/12) without glasses were tested with glasses to determine whether their near vision could be improved. The power of the glasses prescribed was based entirely on the participant’s unaided near visual acuity: glasses of power 1.0D, 1.5D, 2.0D, 2.5D and 3.0D were prescribed to subjects with an unaided near visual acuity of 1.2M, 1.6M, 2.5M, 4.0M and <4.0M respectively. Subjects were considered presbyopic if they had an unaided near visual acuity of 1.2M or poorer at 40 cm, correctable with spherical (non-astigmatic) near glasses to 0.8M or better.

### Questionnaires

Immediately after the vision assessment, trained non-medical study personnel administered a home-based oral questionnaire to persons meeting the above visual and glasses criteria to gather data on age, sex, education level and literacy, household size, primary occupation, engagement in income-producing work, self-reported limitations to work due to poor vision and self-reported savings over the last 6 months. Baseline information on income and NVRQOL (see Assessment of Outcomes, below) was also recorded. A follow-up home survey in March 2018 (8 months later) gathered information on income, NVRQOL and self-reported frequency of glasses wear among both study groups. Both baseline and follow-up data were collected digitally using CommCare HQ software (Dimagi, Inc., Cambridge, MA, USA).

Additionally, an ophthalmologist (NC), optometrist (VFC) and non-medical person resident in the region (PR) independently rated all primary occupations reported by participants as most, moderately or least-visually demanding. Differences between raters were adjudicated by consensus at a single face-to-face meeting.

### Assessment of outcomes and masking

The primary trial outcome was the between-group difference in change in monthly income (self-reported in Bangladeshi taka over the past 4 weeks) between baseline and follow-up surveys. Investigators collecting this information were unaware of participant group assignment. The secondary outcome was the between-group difference in change in NVRQOL between baseline and endline, based on an 8-question version of the instrument described by Patel et al. (minimum [best] score 8, maximum [worst] score 40) [10].

### Sample size

The primary outcome, change in income, is a non-normally distributed parameter better compared using proportions falling within various quartiles for sample size calculations. With a two-sided significance level of p = 0.05 and power of 80%, to detect a 10% between-group difference in the proportion in the highest quartile of income (35% versus 25%), corresponding to an odds ratio of 1.6, at least 349 participants were needed in each group. Assuming a 15% rate of attrition based on previous studies in this population, a total sample of 822 persons (411 in each group) was required. The sample size was based upon the 3rd quartile of income in the control group. By definition 25% will lie above the 3rd quartile of income in the control group—no matter what the distribution of income in the control group–no matter what the actual 3rd quartile of income is. The hypothesis test is based upon a comparison of proportions in the two groups so we know that 25% lie above the 3rd quartile in the control group, and we hypothesize that in the intervention this will shift to 35% above the 3rd quartile.

### Statistical analysis

Differences in baseline characteristics by study group were analysed using the t-test or Wilcoxon rank-sum test for continuous variables with or without normal distribution respectively, the Chi-squared or Fisher’s exact test (with cell frequency<5) for binary categorical variables, and ordinal logistic regression for ordinal categorical variables. Normality was checked using histograms and Q-Q plots. Change in income (main outcome) was compared between study groups using an intention-to-treat approach. Where data were missing, this appeared to be at random, and so no imputation was carried out. Data for the primary outcome are presented as median and inter-quartile range due to lack of normality. Changes in self-reported monthly income were categorized by quartiles and the unadjusted and adjusted intervention effects, odds ratio, and 95% CI were estimated using ordinal logistic regression. A major assumption underlying ordinal logistic regression is that the relationship between each pair of outcome groups is the same, the proportional odds assumption or the parallel regression assumption [11]. The omodel test showed that this study fulfilled the proportional odds assumption.

Unadjusted and adjusted mean between-group differences were estimated for the secondary outcome NVRQOL using linear regression. A histogram of residuals and scatterplots of residuals against fitted values and model variables did not reveal any obvious violation of the regression assumptions. All variables with P<0.05 in the simple regression analysis were included in multiple regression models. All statistical analyses were performed using a commercially available software package (Stata 15.1, StataCorp, College Station TX, USA).

## Results

A total of 10,884 participants aged ≥35 and ≤65 years were identified and examined during the baseline census. Among these, 7229 (66.4%) were excluded during the eye examination for lack of presbyopia, poor distance vision in either eye or ownership of glasses (See Fig 1 Study Flowchart for details). Among 3655 (33.6%) persons meeting vision/glasses criteria, 863 (23.6%) had near vision-intensive jobs and were eligible for the trial, but 39 (4.52%) were not at home during return visits. A total of 824 (95.5%) persons were randomly assigned 1:1 to intervention (n = 423, 51.3%) or control (n = 401, 48.7%). Among these, all received their allocated intervention, and 8-month follow-up was completed by 93.4% (395/423) and 96.8% (388/401) of the intervention and control groups, respectively.

At baseline, the study groups did not differ significantly with regard to major variables: the mean age was approximately 47 years for both groups, about a third were literate, some 80% were currently engaged in income-producing work, about a half in occupations rated as “most near vision-intensive,” and the power of reading glasses provided was modest, a median of only 1D in each group (Table 1). The three most common self-reported primary occupations at baseline were agriculture farming (n = 124, 15.0%), weaver (n = 110, 13.3%) and grocer (n = 76, 9.22%), with livestock farming, handicraft work, and tailor being common jobs. Eight months later, self-reported use of reading glasses was 88.3% (347/393) and 7.81% (10/128) (P<0.001, Chi-square) in the intervention and control groups respectively. In the intervention group, 57.3% of those who responded (225/393) reported wearing study glasses at least 4 days/week. No participants reported eye strain, injury from broken spectacles or other harm related to the intervention.

**Table 1:** Baseline characteristics of participants by study groups.

Baseline characteristics	Intervention Group (n = 423)	Control Group (n = 401)
**Age, Mean (SD), y** ^a^	46.9(7.73)	47.4 (7.51)
**Male sex, n (%)** ^b^	215 (50.8)	211 (52.6)
**Literate, n (%)** ^b^	145 (34.3)	146 (36.4)
**Household size, Median (IQR)** ^c^	4(4–6)	5 (4–6)
**Engaged in income-producing work, n (%)** ^b^	352 (83.2)	330 (82.3)
**Spherical equivalent power of near correction (diopters, same in both eyes), Median (IQR), D** ^c^	1 (1–1.5)	1 (1–1.5) ^e^
**Rating of visual demanding of primary occupation at baseline, n (%)** ^d^		
Least visually demanding	11 (2.60)	11 (2.74)
Moderately visually demanding	189 (44.7)	161 (40.2)
Most visually demanding	223 (52.7)	229(57.1)
**Reports limiting work due to vision, n (%)** ^d^		
Not at all	50 (11.8)	32 (7.98)
Rarely	115(27.2)	128 (31.9)
Some of the time	191(45.2)	188 (46.9)
Most of the time	62(14.7)	52 (13.0)
All of the time	5 (1.18)	1 (0.25)
**Reports saving money in the last 6 months, n (%)** ^b^	152 (35.9)	119 (29.7)

Abbreviation: SD, standardized deviation; IQR, inter quartile range

^a^ T-test;

^b^ Chi-squared test;

^c^ Wilcoxon rank sum test;

^d^ Ordinal logistic regression;

^e^ one missing value

At baseline, there was no significant difference in self-reported median monthly income between the intervention (US$35.3) and Control (US$35.3) groups (P = 0.59). At endline eight months later, the median income values were US$47.1 and US$35.3 respectively, representing a 33.4% greater increase in the intervention compared to the control group. Participants in the intervention group were 1.38 times as likely to be in a higher quartile of income change compared to the control group (95% CI 1.06, 1.78, P = 0.02, logistic ordinal regression). The unadjusted change in NVRQOL was also 15% higher in the intervention group (5.99 points on a 40-point scale, 95% CI 4.96, 7.03, P<0.001, linear regression) (Table 2).

**Table 2:** Effect of randomization group on change in primary outcome (self-reported monthly income) and secondary outcome (total score on near vision related quality of life [NVRQOL] form) from baseline.

	**N**		**Baseline**		**Endline**		**Difference (95%CI)**	**P**
	**Intervention**	**Control**	**Intervention**	**Control** ^a^	**Intervention**	**Control**	**Odds Ratio**	
**Self-reported monthly income** **Median (IQR), USD**	423	401	$35.3 ^b^ (5.88–76.5)	$35.3 ^c^ (10.6–76.5)	$47.1 ^d^ (4.71–105.9)	$35.3 ^e^ (2.35–94.1)	1.38 (1.06, 1.78)	0.015^f^
	**Intervention**	**Control**	**Intervention**	**Control** ^g^	**Intervention**	**Control**	**Unadjusted between-group difference in change (95%CI)**	**P**
**NVRQOL score** **(Higher indicates better QOL), Mean (SD)**	423	401	36.5 (6.08)	36.9(5.64)	43.5 (5.64) ^h^	38.0 (6.34) ^i^	5.99 (4.96, 7.03)	<0.001^j^

Abbreviation: IQR, inter quartile range; SD, standard deviation; 1USD = 85 Bangladeshi taka; NVRQOL, Near vision related quality of life; CI, Confidence interval

^a^ Wilcoxon rank sum test;

^b^ Thirteen missing values;

^c^ Eight missing values;

^d^ Thirty-four missing values;

^e^ Nineteen missing values;

^f^ Ordinal logistic regression, self-reported monthly income changes from baseline categorized by quartiles.

^g^ Two-sample t test;

^h^ Twenty-eight missing values;

^i^ Thirteen missing values;

^j^ Linear regression

In multiple ordinal logistic regression models, predictors of greater change in self-reported monthly income included allocation to the intervention group (OR 1.45, 95% CI 1.12, 1.88, P = 0.005), male sex (OR 2.41, 95% CI 1.84, 3.16, P <0.001) and not engaging in income-producing work at baseline (OR 2.35, 95% CI 1.69,3.26, P<0.001) (Table 3). In multiple linear regression models, predictors of greater improvement in NVRQOL included membership in the intervention group (Beta = 6.04, 95% CI 5.06, 7.02, P<0.001) and engaging in income-producing work at baseline (Beta = 1.99, 95% CI 0.67, 3.32, P = 0.003). A positive dose-response relationship was generally apparent between greater self-reported limitations in work at baseline and larger improvement in NVRQOL (Table 4).

**Table 3:** Ordinal logistic regression model of potential predictors of change in self-reported monthly income categorized by quartiles.

Variables	N = 824			
	Simple regression		Multiple regression ^a^	
	OR (95% CI)	P-value	OR (95% CI)	P-value
**Membership in the intervention group**	**1.38 (1.06, 1.78)**	**0.02**	**1.45 (1.12, 1.88)**	**0.005**
**Age (years)**	1.01 (0.99, 1.02)	0.24		
**Male sex**	**2.09 (1.61, 2.72)**	**<0.001**	**2.41 (1.84, 3.16)**	**<0.001**
**Literate**	1.08 (0.82, 1.41)	0.59		
**Average household size**	1.01 (0.94, 1.08)	0.77		
**Spherical equivalent of near correction (diopters)**	0.90 (0.64, 1.25)	0.53		
**Not engaged in income-producing work at baseline**	**1.96 (1.42, 2.70)**	**<0.001**	**2.35 (1.69, 3.26)**	**<0.001**
**Rating of visual demand of primary occupation at baseline, n (%)**				
Least visually demanding	**Reference**			
Moderately visually demanding	0.59 (0.26, 1.34)	0.21		
Most visually demanding	0.66 (0.29, 1.52)	0.33		
**Self-reports limiting work due to vision at baseline**				
Not at all	Reference			
Rarely	1.02 (0.62, 1.68)	0.93		
Some of the time	1.14(0.71, 1.84)	0.57		
Most of the time	0.69 (0.40, 1.20)	0.19		
All of the time	2.21 (0.51, 9.55)	0.29		
**Reports saving money in the last 6 months at baseline**	1.06 (0.81, 1.39)	0.66		

Abbreviation: OR, odds ratio; CI, confidence interval

^a^ All variables with P<0.05 in the simple regression analysis were included in the multiple regression model.

**Table 4:** Linear regression model of potential predictors of change in near vision related quality of life (NVRQOL) from baseline (higher score indicates greater improvement).

Variables	n = 824			
	Simple regression		Multiple regression ^a^	
	β (95% CI)	P-value	β (95% CI)	P-value
**Membership in the intervention group**	**5.99 (4.95, 7.03)**	**<0.001**	**6.04 (5.06, 7.02)**	**<0.001**
**Age (years)**	0.01 (-0.07, 0.08)	0.86		
**Female sex**	-0.26 (-1.38, 0.86)	0.64		
**Literate**	-0.34 (-1.51, 0.82)	0.56		
**Average household size**	0.16 (-0.11, 0.43)	0.24		
**Spherical equivalent of near correction (diopters)**	**2.50 (0.90, 4.11)**	**0.002**	1.36 (-0.01, 2.73)	0.052
**Not engaged in income-producing work at baseline**	**1.98 (0.57, 3.39)**	**0.006**	**1.99 (0.67, 3.32)**	**0.003**
**Rating of near vision intensive primary occupation at baseline, n (%)**				
Least near vision intensive	Reference			
Moderately near vision intensive	-0.06 (-2.59, 2.48)	0.97		
Most near vision intensive	1.00 (-1.47, 3.48)	0.43		
**Reports limiting work due to vision at baseline**				
Not at all	Reference		Reference	
Rarely	**2.44 (0.92, 3.96)**	**0.002**	**2.92 (1.49, 4.36)**	**<0.001**
Some of the time	**5.37 (3.93, 6.83)**	**<0.001**	**5.69 (4.30, 7.08)**	**<0.001**
Most of the time	**8.25 (6.13, 10.4)**	**<0.001**	**8.12 (6.13, 10.11)**	**<0.001**
All of the time	**7.42 (0.94, 13.9)**	**0.025**	**5.07(0.08, 10.06)**	**0.047**
**Reports saving money in the last 6 months at baseline**	0.84 (-0.35, 2.04)	0.17		

Abbreviation: **β**, coefficient estimate; CI, confidence interval

^a^ All variables with P<0.05 in simple regression analysis were included in the multiple regression analysis.

## Discussion

Consistent with the significant increase in productivity observed in our previous PROSPER trial of reading glasses among Indian tea pickers [8], the THRIVE trial’s cohort of labourers from a range of different occupations had significantly-increased self-reported income 8 months after receiving spectacles, compared to controls. Both studies found that treatment of only modest presbyopia (median glasses power of 1D in the current report) yielded significant impact, and further observed that such levels of presbyopia were highly-prevalent in working-age populations, including some 50% of those aged 35–65 years undergoing near vision testing in THRIVE. Advantages of the current study over our earlier PROSPER trial are the inclusion of a broad range of occupations and direct assessment of their income.

It has been suggested that increasing workforce participation and productivity among women results in faster economic growth [12]. It is thus relevant to both the SDG of poverty alleviation and to SDG 5 of gender equality that half of participants in THRIVE were women. However, men did have a significantly greater likelihood of increased income in THRIVE, as did those who were not engaged in economically-productive work at baseline. This latter finding holds out the encouraging possibility of potentiating income gains among those who are currently not gainfully employed, if cost-effective and sustainable community-based strategies can be devised to identify those with presbyopia. Our regression models found no impact on income gain from our visual-intensity variable, possibly because the cohort was recruited to consist largely of those working in near vision-intensive jobs, or because of inaccuracies in our investigator assessment of the visual requirement of various occupations.

Considering the practical implications of the THRIVE and PROSPER trials, workplace screening of large cohorts in a single setting, as in PROSPER, offers an efficient model and may also be sustained by companies eager to reap gains in productivity. Community-based programs of reading glasses provision conducted by non-medical personnel, as in THRIVE, allow for broad access and cost recovery through glasses sales. This model has been practiced widely by the non-governmental organisations VisionSpring and BRAC [13], partners in this study. Use of lay personnel rather than medical professionals to fit and dispense reading glasses is consistent with regulations permitting over-the-counter sales of such devices in countries including the United States [14, 15]. Ironically, over-the-counter sales of near vision corrections appear more likely to be permitted in wealthy countries than in LMICs, where spectacle coverage is lowest [4, 5]. It is likely that workplace and community-based glasses distribution strategies are complementary.

We recently carried out a review of trials on health strategies to improve productivity and income [8]. Our conclusions remain unchanged that spectacle provision compares very favourably with the few other existing trial-tested interventions, including distribution of insecticide-treated mosquito nets [16] and calorie [17] and iron supplementation [18–21], in terms of impact, uptake and potential for sustainability. No other health-related trial has reported an effect size greater than 15% for productivity or income, and many results have been negative [17–19, 21]. Acceptance of study glasses was very high in THRIVE, nearly 90%. Reports have documented high rates of glasses retention over the medium term [7, 22], which may be contrasted with the supply-chain and compliance challenges inherent in regular delivery of nutritional supplements.

Findings of THRIVE are consistent with studies of other ocular interventions documenting direct economic benefits after adult cataract surgery [23] and economically-important educational advantages from glasses distribution to school-going children [24]. In terms of their scalability as strategies for large-scale poverty alleviation, both cataract surgery [25] and refraction for distance glasses [26] require significant training and have been associated with poor results [25, 26] in low-resource areas. Results of THRIVE suggest that distribution strategies implemented by non-medical personnel are practical for reading glasses, making them particularly attractive in low-resource settings.

Strengths of the THRIVE trial include its randomised, controlled design, wide-based sampling strategy (covering 15 of Bangladesh’s 64 districts), and high rates of follow-up. Weaknesses must also be acknowledged: the trial’s main and secondary outcomes were both self-reported, and the investigators did not feel that participant masking with sham glasses was ethical in this setting. Thus, the potential for placebo effects cannot be excluded. Workplace-based trials may offer the advantage of objective outcome assessment by employers independent of investigators. It is important however to determine whether results of trials such as PROSPER can be extended to the very large number of artisans and other labourers working outside of organised corporate structures. Given the informal economy within which such persons work and are paid, it will be challenging to devise objective indicators to replace self-reported income. Taken within the context of the findings of PROSPER, THRIVE provides further evidence that low-cost provision of reading glasses may yield measurable economic gains in low-resource settings.

Future trials are needed to extend the evidence base supporting glasses distribution to other work settings, such as factories, and to assess their impact on long-term workplace retention among older workers. Additional related areas of strategic importance include measuring the impact of reading glasses on adult literacy programs and advocacy to promote the legalisation of over-the-counter sales of reading glasses, reducing the potential bottleneck posed by limited numbers of trained vision providers in LMICs.

## Supporting information

S1 Checklist CONSORT 2010 checklist of information to include when reporting a randomised trial*. (PDF)

S1 File (PDF)

S2 File (PDF)

S3 File (JPG)

S4 File Registered protocol. (PDF)
