# Supplementary material for: The effect on income of providing near vision correction to workers in Bangladesh: The THRIVE (Tradespeople and Hand-workers Rural Initiative for a Vision-enhanced Economy) randomized controlled trial
Source: PLoS One. 2024 Apr 3;19(4):e0296115. doi: 10.1371/journal.pone.0296115 (PMC10990163; doi:10.1371/journal.pone.0296115)
Supplement: S1 File — (PDF) [file pone.0296115.s002.pdf]

Question Can provision of free reading glasses for presbyopia correction increase the income of various visually-demanding occupations in Bangladesh?
Findings This individual randomized controlled trial of 824 participants showed that participants in the intervention group were significantly (OR 1.38, 95% CI 1.06, 1.78, P= 0.015) more likely to have greater gains in self-reported income compared with controls 8 months after receiving spectacles.
Meaning Provision of reading glasses has potential as a poverty-alleviation strategy in this setting.

Registry ClinicalTrial.gov
Study Registration Number NCT03719196
