# Supplementary material for: The effect on income of providing near vision correction to workers in Bangladesh: The THRIVE (Tradespeople and Hand-workers Rural Initiative for a Vision-enhanced Economy) randomized controlled trial
Source: PLoS One. 2024 Apr 3;19(4):e0296115. doi: 10.1371/journal.pone.0296115 (PMC10990163; doi:10.1371/journal.pone.0296115)
Supplement: S2 File — (PDF) [file pone.0296115.s003.pdf]

Supplemental Document
THRIVE Study
July 29, 2022

OCCUPATION CATEGORIES

**Occupation types**	**Visually demanding categories**	**Frequency**
Carpenter/Bamboo, Cottage products maker/tube well maker/packet maker	Most visually demanding	41
Electrician/Mechanic/welding work	Most visually demanding	7
Garment, tailor, weaver, handicrafts worker, bags, mats	Most visually demanding	297
Goldsmith worker/Remittance/Barber/Kumar	Most visually demanding	17
Government/medical & paramedical/teacher/tutors/NGOs	Most visually demanding	8
Works at shop	Most visually demanding	80
Agriculture, and animal husbandry	Moderate visually demanding	253
Businessman/Hawker	Moderate visually demanding	18
Driver-Bus/Truck/Taxi/Passenger vehicle/Boatman	Moderate visually demanding	29
Private job	Moderate visually demanding	10
Motor car mechanic/mason/soil clipper/other industrial laborer	Moderate visually demanding	39
Painter	Moderate visually demanding	2
Daily wage labor	Least visually demanding	15
Maid	Least visually demanding	5
Land leased out	Least visually demanding	1
Old age pension/widow pension/aid for the disadvantaged	Least visually demanding	2
**Total**		**824**
