# Supplementary figures and images for: The effect on income of providing near vision correction to workers in Bangladesh: The THRIVE (Tradespeople and Hand-workers Rural Initiative for a Vision-enhanced Economy) randomized controlled trial
Source: PLoS One. 2024 Apr 3;19(4):e0296115. doi: 10.1371/journal.pone.0296115 (PMC10990163; doi:10.1371/journal.pone.0296115)

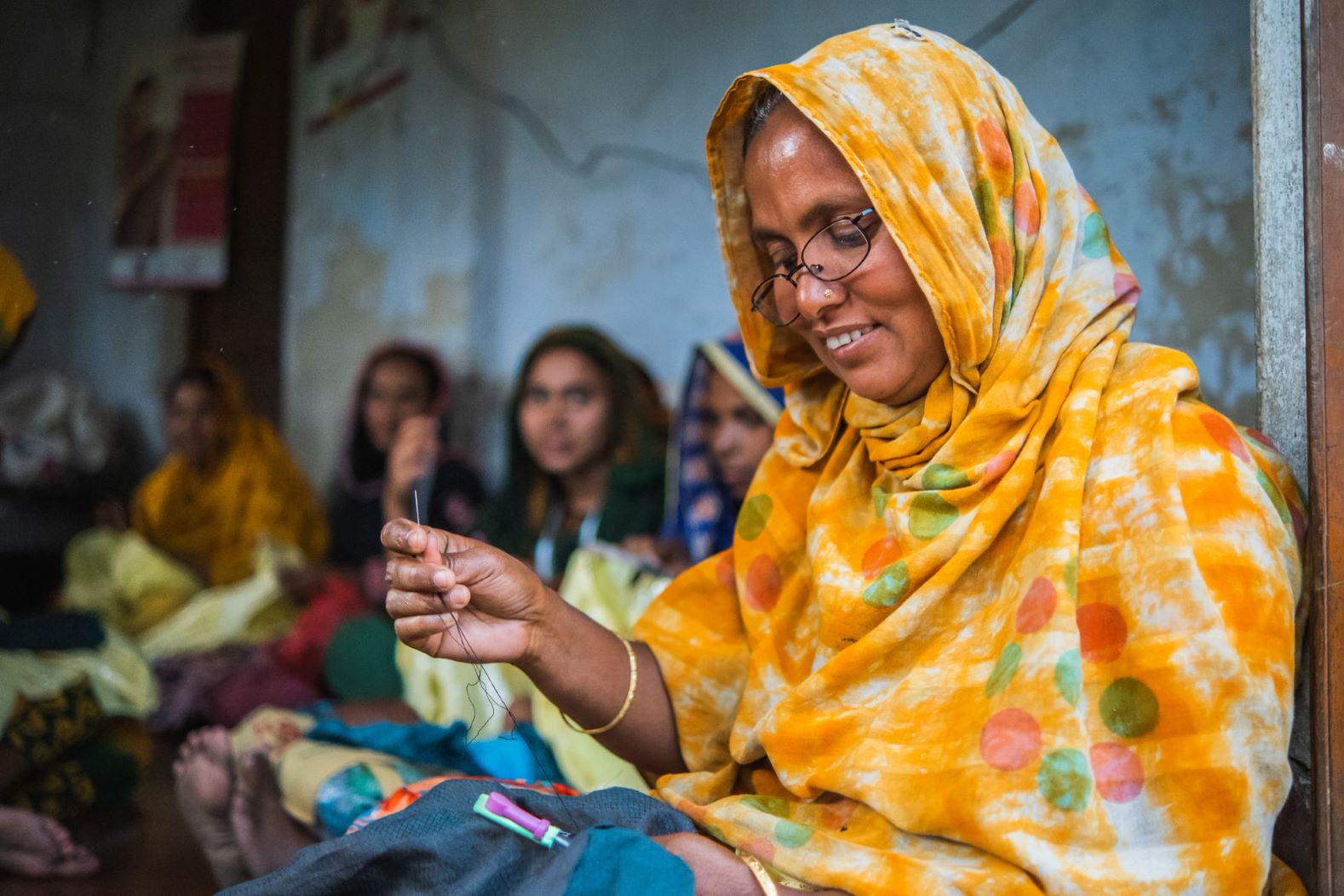

Supplement: S3 File — (JPG) [file pone.0296115.s004.jpg]
