# Supplementary material for: The effect on income of providing near vision correction to workers in Bangladesh: The THRIVE (Tradespeople and Hand-workers Rural Initiative for a Vision-enhanced Economy) randomized controlled trial
Source: PLoS One. 2024 Apr 3;19(4):e0296115. doi: 10.1371/journal.pone.0296115 (PMC10990163; doi:10.1371/journal.pone.0296115)
Supplement: S4 File — (PDF) [file pone.0296115.s005.pdf]

Registered Protocol
THRIVE

Brief Summary:

The issue of uncorrected Presbyopia is an emerging health and livelihoods problem in Bangladesh. Presbyopia is easily corrected with an appropriate pair of reading glasses. Non-governmental organizations and government agencies have been working in many emerging markets, including Bangladesh, to increase eyeglass coverage among low-income adults by introducing basic vision screening and the dispensing of reading glasses at the community level. While vision screening is typically considered a health intervention, the primary motivation of these organizations and government agencies is to correct blurry vision with the intention of improving adults' income earning potential.

VisionSpring and BRAC (previously known as Bangladesh Rural Advancement Committee) are two such NGOs who are partnering to implement a "Reading Glasses for Improved Livelihoods" program. VisionSpring is a social enterprise with a mission to increase access to affordable eye care and glasses in low-income settings. BRAC is an international development organization dedicated to alleviating poverty by empowering the poor.

VisionSpring and BRAC have initiated a Randomized Control Trial to assess the economic and social impact that reading glasses may have on individuals that earn less than $4 a day. The study hypothesizes that post-intervention, the adoption of reading glasses will increase productivity related to paid work, and will enhance the vision-related quality of life of adults with Presbyopia.

This experimental trial is designed to test the aforementioned hypotheses. In March 2017 a census survey was conducted in 59 villages of adults aged ≥35 and ≤65 years old in order to identify individuals with uncorrected presbyopia who would be eligible for enrollment in the study intervention. Following household visual acuity screenings, study participants were selected for enrollment. Randomization was conducted at the household level. Based on the inclusion criteria, a total of 824 random households were surveyed at the baseline. Following completion of the baseline survey, in August 2017 the treatment group was given reading glasses free-of-cost. The endline survey will be conducted in March 2018, eight months from the baseline. Although some income-related outcomes may take more time to manifest, it is anticipated that the wearing of glasses precipitates changes in a relatively short time frame. The control group will receive reading glasses at the conclusion of the endline survey.

Study Type :	Interventional (Clinical Trial)
Estimated Enrollment :	824 participants
Allocation:	Randomized
Intervention Model:	Parallel Assignment
Masking:	Investigator-masked
Primary Purpose:	Other
Official Title:	Assessment of the Impact of Reading Glasses on Livelihood and Quality of Life in the Context of Rural Bangladesh (THRIVE)

Intervention:

Experimental Arm: Reading glass provided free of cost based on assessment by trained community workers.

Control: Provision of identical reading glasses at the end of an 8-month observation period

Outcomes:

Primary outcome measure: Income (Bangladeshi Taka) [Time Frame: 7 to 8 months.] Each participant's income data will be captured before and after the intervention by structured questionnaire (e.g: how much taka you earned in last 30 days?). The data will then be calculated before and after the intervention (8 months period) and will be compared between intervention and control group participants to see the difference.

Secondary outcome: Near vision-related quality of life, including the following domains:

- Difficulties in performing daily wise task: [Time Frame: 7 to 8 months.]. Likert scale (scale of 5) will be used to capture data on participants' Difficulties in performing daily wise tasks. Each question related to visual function is rated on a five-pointed scale consisting of (1) None; (2) Mild difficulty; (3) Moderate difficulty; (4) Severe difficulty; (5) Extreme difficulty.
- Quality of vision: Likert scale [Time Frame: 7 to 8 months.] Self-reported quality of vision as per responses of the respondents will be measured. Measurement will be based on a five point Likert scale as (1) Very Good; (2) Good; (3) Moderate; (4) Bad; (5) Very Bad.
- Discomfort in functioning social activities [Time Frame: 7 to 8 months.] Social functioning such as feel ashamed or discomfort due to eye problem in the society will be measured. Measurement will be based on a five point Likert scale consisting of (1) Never; (2) rarely; (3) Sometimes; (4) Often; (5) Very often.

Eligibility criteria:

Ages Eligible for Study:	35 Years to 65 Years (Adult, Older Adult)
Sexes Eligible for Study:	All
Accepts Healthy Volunteers:	No

Inclusion Criteria:

- 35-65 years of age (inclusive)
- Presbyopia present (Distance vision >= 6/12 in both eyes, with inability to see the 6/13 line at 40 cm with both eyes, correctable with near glasses)
- Has never used reading glass or any other eyeglasses
- Selective occupations associated with the need for clear vision at near distances (tailor, barber, self-employed artisan, etc.)
- Willing to provide contact information and give informed consent
- Exclusion Criteria:
- Serious medical/mental and or eye condition, and
- Meets criteria but does not wish to participate

Detailed Description:

BRAC (previously known as Bangladesh Rural Advancement Committee) has conducted a study on the Assessment of the Impact of Reading Glasses on Livelihoods and Quality of Life in the Context of Rural Bangladesh in 2017 with financial and technical assistance from VisionSpring. The goal is to assess the impact of reading glasses on economic return and social angles for people in rural areas in Bangladesh.

BRAC and VisionSpring have developed an experimental trial to test the effects of reading glasses. The study will utilize both pre and post-intervention data to identify the causal effects. A randomized control trial is considered to be the most rigorous method of determining whether a causal relationship exists between an intervention and outcome. This is a Randomized Control Trial (RCT) conducted at the household level.

First, investigators selected 59 villages in Bangladesh where the BRAC/VisionSpring distribution of reading glasses has not been present. A census survey was carried out in the 59 villages to identify those suffering from Presbyopia who were eligible for glasses.

Second, participants were recruited through a household Presbyopia screening of individuals over 35 years old and less than 65 years old. Participants with chronic eye diseases that require regular and intensive treatment were excluded from the study.

A structured questionnaire was used at the census survey where demographic information, income, and occupation were asked. Randomization was taken place after the census survey, and a study sample was selected based on the inclusion criteria of the study which are: (1) Over 35 years of age, (2) Presbyopia positive, (3) Have never used reading glasses, (4) Selective occupation (tailor, barber, self-employed artisan etc), (5) Willing to provide contact information and give informed consent.

The study proposal defines two rounds of data collection to be conducted; baseline data collection was completed in August 2017 and follow-up data will be conducted in March 2018. A quantitative questionnaire was distributed at the baseline survey in order to understand the livelihoods and quality of life before the intervention began. Skilled interviewers were recruited for this study, and a three-day intensive training was held to reinforce the goals of the study. The training module was developed in Bangla to guide the interviewers in the field. Questions related to socio-demographic characteristics, economic status of the household (e.g. monthly income), the productivity of the respondent and quality of life were collected during baseline, and the same questionnaire will be used in the baseline survey.

A multi-stage simple random sampling technique has been used in this study. The sample size of the study is pre-determined through a power calculation, the inclusion criteria of being a Presbyopic patient. As our primary outcome is income, a non-normally distributed parameter, it is better to compare proportions falling in various quartiles for sample size calculations. With a two-sided significance level of $p=0{\cdot}05$ and power of 80%, to detect a 10% between-group difference in the proportion in the highest quartile of income (35% versus 25%), corresponding to an odds ratio of 1.6, 349 participants would be needed in each group. Assuming a 15% rate of attrition based on previous studies in this population, a total sample of 822 persons (411 in each group) would be required. At the baseline, a total of 824 random households have been surveyed (T=423; C=401).

As the study is randomized at the individual level, there is a possibility to have the spillover effect. To minimize the spillover effect, one participant was selected from each household. There is a risk that members of the control group may see the benefits experienced by others in their community and buy a pair of reading glasses during the study. In an attempt to mitigate this risk, members of the control group were promised free glasses at the end of the study.

Investigators will use a difference-in-difference analysis to estimate the impact of the reading glasses study based on self-reported monthly income (primary outcome), and near vision-related quality of life.

To measure the quality of life, investigators will use a standardised instrument to look at respondents' perceptions of their facility with caregiving, household work, ability to (for those who are literate), mobile phone use, personal hygiene, and dependence.
